# State anxiety and emotional face recognition in healthy volunteers

**DOI:** 10.1098/rsos.160855

**Published:** 2017-05-31

**Authors:** Angela S. Attwood, Kayleigh E. Easey, Michael N. Dalili, Andrew L. Skinner, Andy Woods, Lana Crick, Elizabeth Ilett, Ian S. Penton-Voak, Marcus R. Munafò

**Affiliations:** 1 MRC Integrative Epidemiology Unit, School of Experimental Psychology, University of Bristol, Bristol, UK; 2 UK Centre for Tobacco and Alcohol Studies, School of Experimental Psychology, University of Bristol, Bristol, UK

**Keywords:** anxiety, emotional face processing, emotion recognition, interpretation bias, 7.5% carbon dioxide

## Abstract

High trait anxiety has been associated with detriments in emotional face processing. By contrast, relatively little is known about the effects of *state* anxiety on emotional face processing. We investigated the effects of state anxiety on recognition of emotional expressions (anger, sadness, surprise, disgust, fear and happiness) experimentally, using the 7.5% carbon dioxide (CO_2_) model to induce state anxiety, and in a large observational study. The experimental studies indicated reduced global (rather than emotion-specific) emotion recognition accuracy and increased interpretation bias (a tendency to perceive anger over happiness) when state anxiety was heightened. The observational study confirmed that higher state anxiety is associated with poorer emotion recognition, and indicated that negative effects of trait anxiety are negated when controlling for state anxiety, suggesting a mediating effect of state anxiety. These findings may have implications for anxiety disorders, which are characterized by increased frequency, intensity or duration of state anxious episodes.

## 1. Background

Emotional face processing is one of a number of fundamental non-verbal components of social interaction [1,2]. Emotional expressions are a rich source of information that enables the viewer to infer the thoughts, emotional state and intention of others, and they can influence behavioural tendencies to approach or avoid others [3,4]. Aberrant emotional face processing has been reported in a number of psychiatric disorders and among individuals with antisocial tendencies [5–9]. In addition, high levels of trait anxiety have been associated with altered emotional processing [10–12], often characterized by a sensitivity to fearful faces. While an ability to quickly identify danger may have adaptive value, this may lead to detrimental consequences if dysregulated. The importance of emotional face processing has been exemplified by a recent model of psychiatric disorder, which suggests that impaired emotional face processing may not just be a symptom, but also a causal factor in the initiation and maintenance of disorder [13]. This model suggests that aberrant emotional face processing (such as a tendency to perceive negativity in faces) leads to inappropriately negative behaviour from the viewer (e.g. aggression, avoidance). This in turn leads to a negative cycle of social interaction, with the person being observed actually displaying negative social behaviour and facial expressions, thereby confirming the viewer's original misperception. Understanding these processes is therefore crucial for understanding the aetiology of psychiatric disorders and developing effective treatment strategies.

Cognitive models of emotional disorders characterize anxiety as hyper-vigilance to threat [14]. This includes selective attention towards, and difficulty disengaging from, threat information [15–18]. In addition, high trait and clinical anxiety have been associated with impairment in the recognition of emotional expressions, although there is question as to whether this impairment is global or emotion-specific. Findings from a meta-analysis suggested global impairment in emotion recognition among adults with clinical levels of anxiety [10], but analyses were not conducted on individual emotional expressions. There have been reports of superior recognition of fearful faces in individuals with high trait anxiety [19,20], although these findings have not been consistently replicated [21,22]. A difference between studies that may contribute to inconsistent findings is whether the study measures accuracy or sensitivity, where the latter takes account of errors (i.e. false alarms). A *bias* towards fear would manifest as a higher hit rate *and* a higher false alarm rate. If only hit rate is reported, the findings would suggest superior processing of fear in anxious samples. However, if sensitivity were analysed, the high false alarm rate would contribute to a low overall sensitivity score and the interpretation would be of an impairment in fear processing in line with a processing *bias*. Therefore, it is important to consider the outcome measure used in individual studies and the nature of errors when interpreting these findings.

Interpretation biases have also been reported in individuals with anxiety [23,24]. For example, social anxiety is associated with a tendency to interpret information negatively (particularly social information). Studies that have explicitly examined interpretation of ambiguous emotional expressions largely support a negative or threat-related interpretation bias, with increased tendency to perceive anger [25,26] and contempt [27]. There is also evidence of increased neural response to angry faces in generalized anxiety disorder [28], which may reflect biases that favour the detection of threat [14].

By contrast, relatively few studies have experimentally investigated how state anxiety affects emotional face processing. This is an important line of investigation as more frequent and intense episodes of state anxiety are a core component of anxiety disorders. Emotional processing theory identifies experience and learned maladaptive associations as key elements of the fear response [29,30], and therefore, it is plausible that state anxiety alone (i.e. in the absence of a learned or trait-like disposition towards anxious avoidance) may induce different patterns of emotional responding than trait anxiety.

Some studies using a threat of electric shock procedure [31] to induce state anxiety have reported similar fear-specific effects. These include greater identification accuracy [32], increased prefrontal–amygdala connectivity [33] and increased startle reactivity [34] during the processing of fearful (compared with happy or neutral) facial stimuli. Other psychological stressors include the Trier Social Stress Test (TSST), although their use has been limited in the area of emotional processing. One study using this task reported an attentional bias to angry faces but only when images were presented to the left visual hemisphere [35]. These paradigms have methodological limitations. For example, it is difficult to administer tests when anxiety is at its peak and they are particularly susceptible to response variation based on individual differences. They also model a specific type of cognitively induced or socially induced anxiety, which may not be applicable to generalized anxiety.

To circumvent these issues, inhalation of hypercapnic gases can be used to induce robust but transient increases in state anxiety. This technique involves inhalation of carbon dioxide (CO_2_)-enriched air, and response to this challenge has been used as a trait marker of panic disorder at higher doses [36]. In this study, we use inhalation of 7.5% CO_2_, which reliably increases self-reported anxiety (e.g. worry, tension) and autonomic arousal (e.g. heart rate (HR), blood pressure) [37]. It has been validated as a laboratory model of generalized anxiety disorder [38], which is sensitive to GABAergic anxiolytic compounds. It has also been shown to induce attentional biases characteristic of anxious populations [39].

Compared with inhalation of air, inhalation of air enriched with 7.5% CO_2_ produces marked increases in subjective anxiety and negative mood, as well as HR and blood pressure [37]. This model is a physiological stressor that lacks cognitive (threat of shock) or social (TSST) components of anxiety. This enables us to isolate the physiological aspects of anxiety, as an important first step in understanding the profile of anxiety's effects on emotional face processing.

In studies 1 and 2, we used the 7.5% carbon dioxide model to investigate the effects of state anxiety on emotion recognition and interpretation bias. Study 2 was a direct replication of study 1 that was conducted to determine whether the findings from study 1 were reproducible. In these studies, we used a six alternate forced choice (6AFC) task which presented six emotions (anger, sadness, surprise, disgust, fear and happiness) to measure emotion recognition, and a two alternate forced choice (2AFC) task which presented ambiguous emotional faces (i.e. composite images that were a mix of anger and happiness) to measure interpretation bias for anger. Owing to time constraints of the inhalation, we were only able to administer one version of the 2AFC task. Anger–happiness morphs were chosen as previous studies have reported anger biases following a social stress test [35] and in individuals with social anxiety [25,26]. Study 3 was an observational study run online that investigated the relationship between emotional face processing and self-report measures of state and trait anxiety, using an adapted version of the 6AFC task.

## 2. Study 1

### 2.1. Methods

#### 2.1.1. Participants

Twenty-one healthy volunteers were recruited from staff and students of the University of Bristol and from the local area via participant email lists, posters and word of mouth. Exclusion criteria included pregnancy, recent use of illicit drugs, daily smoking, high caffeine consumption (greater than or equal to 8 drinks per day), recent use of prescribed medication (past eight weeks), asthma, respiratory illness, cardiovascular disease and history of drug/alcohol dependence. Pregnancy and recent drug use were verified by urine screen, while all other criteria were assessed via self-report. Participants were also required to be in good psychiatric health, which was assessed using an adapted version of the MINI-International Neuropsychiatric Interview [40]. Participants were also excluded if they had high blood pressure (greater than 140/90 mmHg), bradycardia (less than 50 beats min^−1^), tachycardia (greater than 90 beats min^−1^) or body mass index (BMI) outside of a healthy range (less than 17 or greater than 30 kg m^−2^), all of which were physically assessed. Participants were asked to refrain from consuming alcohol for 36 h. Expired breath alcohol was measured upon arrival and participants were excluded if their breath alcohol reading was higher than zero. Participants were reimbursed £20 for taking part in the study.

#### 2.1.2. Study design

The study used a within-subjects design with a primary factor of gas (air, 7.5% CO_2_). The analysis of 6AFC task data included an additional six-level factor of emotion (anger, sadness, surprise, disgust, fear and happiness). To assess the subjective and physiological effects of the inhalations, self-report ratings of anxiety and mood, and physiological measurements of HR and blood pressure were taken after each inhalation.

#### 2.1.3. Measures and materials

**Figure 1.**
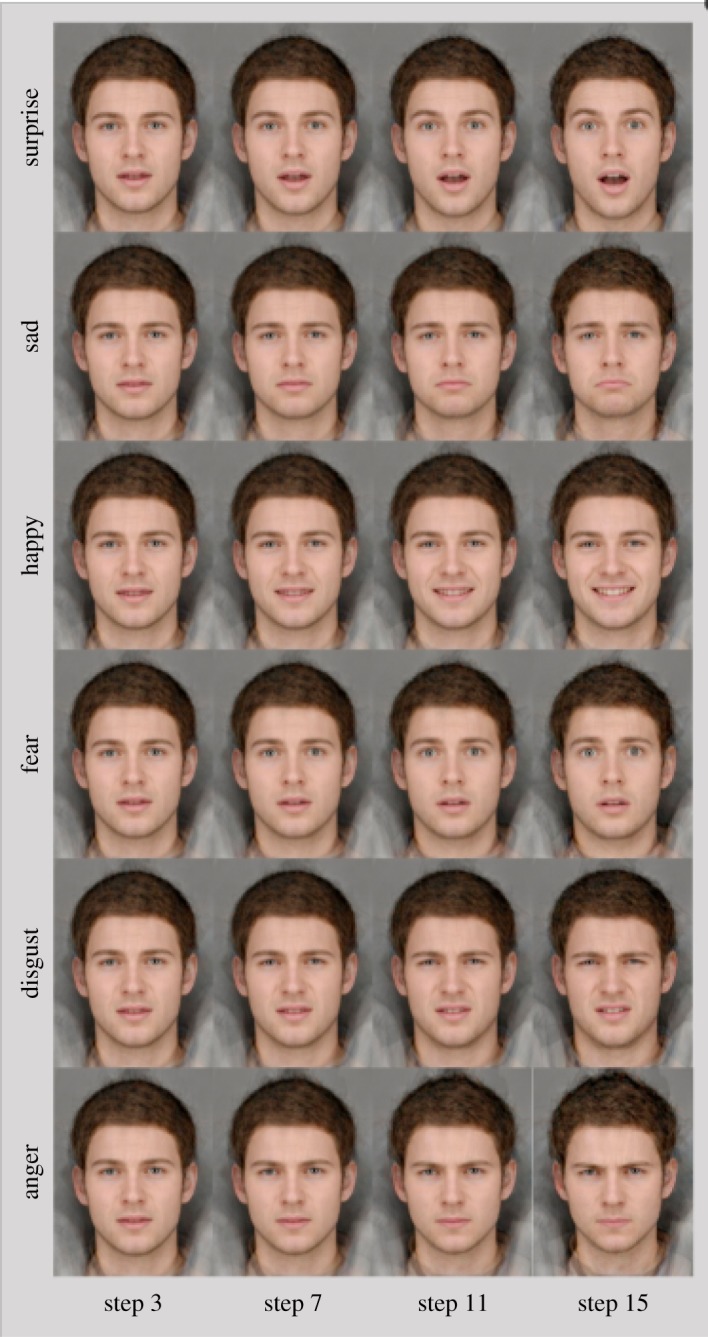
Selected images from the six emotional continua in the 6AFC task.

The 6AFC task comprised 180 trials (30 for each emotion). In each trial, participants were presented with a face displaying one of six emotions: anger, sadness, surprise, disgust, fear and happiness. Prototypical composite images of the six basic facial expressions of emotion were generated from 12 individual male faces showing each of the six expressions. The 12 original images were each delineated with 172 feature points, which allowed both shape and colour information to be averaged across the faces to generate ‘average’ anger, sadness, surprise, disgust, fear, happiness, using established techniques [41]. An overall emotional prototype face was then generated by averaging the exemplars for each emotional expression. A sequence of 15 images was generated for each of the six emotions, equally spaced in emotional intensity from the overall prototype face to the emotional exemplar (figure 1). This results in the strength of the visible emotion increasing across the 15-image sequence, and each image was displayed twice during the task. This stimulus set has been used in a number of published studies [42–47]. Images were presented for 150 ms, followed by a backwards mask (white noise) of 250 ms to avoid after images. Six emotional descriptors (anger, sadness, surprise, disgust, fear and happiness) were then presented on screen. Participants were required to select the descriptor that best described the emotion that was present in the face, using the computer mouse. The descriptors stayed on screen for 10 s or until a response was made. A fixation cross was displayed on screen between images, for between 500 and 1500 ms. The task was delivered using E-Prime Professional v. 2.0 software (Psychology Software Tools Inc., Pittsburgh, PA, USA).

The 2AFC task comprised 45 trials. In each trial, participants were presented with an emotional face stimulus that was a morph of the angry and happy emotional exemplars used in the 6AFC task. The images were part of a morph continuum ranging from a full happiness emotional exemplar to a full anger emotional exemplar. Therefore, the individual images presented on screen varied in the amount of each emotion presented. Fifteen equally spaced images (frames) across this morph sequence were chosen as stimuli. Participants were required to decide whether the face was ‘happy’ or ‘angry’ via designated keys on the keyboard. The image was presented for 150 ms, then backwards masked for 250 ms with a visual noise mask. A response prompt then stayed on screen until a response was made. The primary outcome was a ‘bias’ score that was calculated as a balance point estimate at which the participant was equally likely to perceive happiness or anger. As the spectrum ranged from happy (image 1) to angry (image) 15, lower threshold scores (i.e. 7 or below) indicate greater biases towards seeing angry (i.e. individuals show an earlier change from perceiving happiness to anger). The task was programmed and run using E-Prime Professional software, v. 2.0 (Psychology Software Tools Inc.).

Questionnaire measures included the Spielberger State-Trait Anxiety Inventory (STAI State and STAI Trait) [48], the Positive and Negative Affect Schedule (PANAS) [49], the Anxiety Sensitivity Index (ASI) [50] and the Eysenck Personality Questionnaire Revised (EPQ-R) [51].

Physiological measures of HR, systolic blood pressure (SBP) and diastolic blood pressure (DBP) were taken at baseline and after each inhalation (Omron M6 BP monitor, Omron Healthcare B.V., UK).

The gas mixtures used were 7.5% CO_2_, 21% O_2_, N balance and medical air (21% O_2_, N balance). The gases were administered through an oro-nasal facemask (Hans Rudolph Inc., USA) with the order of gas counterbalanced across participants. For safety reasons, the gas was administered single blind.

#### 2.1.4. Procedure

Prior to the study day, participants completed a telephone screening to assess basic eligibility. On the day of testing, written informed consent was taken and further screening procedures were conducted. These included a urine screen for recent drug use and pregnancy in females, assessment of SBP, DBP, HR, BMI and completion of the neuropsychiatric interview. Baseline questionnaire measures were also completed (STAI State, STAI Trait, PANAS and ASI) prior to the first inhalation. Participants were then fitted with the oro-nasal facemask which was connected to either the 7.5% CO_2_ gas or medical air as per the gas order counterbalancing. During each inhalation, the 6AFC and 2AFC tasks were completed, with task order counterbalanced across participants. Participants inhaled the gas for 60 s before they started the computer tasks to allow anxiety levels to stabilize. The inhalations lasted for the duration of the tasks, which was up to (but no more than) 20 min. After a 30 min rest period, participants completed the alternate inhalation, during which they completed the computer tasks again. Immediately after each inhalation, SBP, DBP and HR were recorded. Participants then completed the state measures of anxiety and mood (STAI State, PANAS) and were instructed to answer retrospectively indicating how they felt when the effect of the gas was at its strongest. After the second inhalation, participants completed the EPQ-R and remained in the room for a further 20 min to allow any effects to dissipate. Participants were fully debriefed as to the nature of the study and given a safety card to keep with them for 24 h. A follow-up call was conducted 24 h later to ascertain if any adverse effects had occurred.

#### 2.1.5. Statistical analysis

All statistical analyses were conducted using IBM SPSS Statistics for Windows (V. 21.0, IBM Corp.). For the 6AFC task, total hits were assessed for outliers using boxplots. Participant data were removed if scores were 1.5 times greater than the interquartile range in both air and 7.5% CO_2_ conditions, or if scores were 3.0 times greater than the interquartile range in one condition. Data were also assessed for normality using skewness and kurtosis statistics. Where Mauchly's Test of Sphericity was *p* < 0.05, Greenhouse–Geisser statistics are reported. Analysis of 6AFC data was conducted in two phases. First, total hits were analysed within a 2 gas (air, 7.5% CO_2_) × 6 emotion (anger, sadness, surprise, disgust, fear and happiness) within-subjects ANOVA (two-sided). The main effect of gas provided an estimate of global emotion recognition ability and the interaction enabled us to explore the emotion-specificity of gas effects. Second, sensitivity scores (unbiased hit rate) were calculated for each emotional expression [52]. We planned to analyse false alarms (as opposed to sensitivity scores) using the same statistical model as used for hit rate data (as described in pre-registered protocol for study 2). However, this was later considered to be inappropriate as false alarms are only meaningful when considered at an emotion-specific level (i.e. are inverse of hits at the global level). Furthermore, they are less informative than sensitivity scores, which balance the ability to accurately identify an emotion (i.e. hits) with erroneous identifications when an emotion is not present (i.e. false alarms). This enables differentiation of whether there is a genuine improvement in the ability to recognize emotion (i.e. recognition accuracy) versus a general tendency to identify it regardless of whether it is present (i.e. bias). This analysis also enables us to identify systematic differences in errors made (in addition to absolute differences in the number of errors) [53]. As emotion-specific sensitivity scores are not independent (i.e. an increase in false alarm rate to one emotion will impact on false alarm rates across other emotions), the ANOVA model was not considered appropriate. Therefore, emotion-specific sensitivity was analysed using six paired-sample *t*-tests. Analysis of 2AFC data comprised a paired samples *t*-test to compare threshold in 7.5% CO_2_ and air conditions (two-sided). To confirm the effect of the state anxiety manipulation, subjective (STAI State, PANAS) and physiological (SBP, DBP, HR) outcomes were compared after 7.5% CO_2_ and air inhalations, using paired sample *t*-tests.

### 2.2. Results

The data that form the basis of the results presented here are available from the University of Bristol Research Data Repository (http://data.bris.ac.uk/data/) (doi:10.5523/bris.tdkhpfjk64ip1o86m4tq9e306).

#### 2.2.1. Characteristics of participants

Participants (*n* = 21; 52% male) were aged between 18 and 22 years (*M* = 20, s.d. = 1). STAI Trait and ASI scores ranged between 23 and 40 (*M =* 32, s.d. = 5) and 2 and 24 (*M* = 12, s.d. = 6), respectively. EPQ-R scores ranged between 0 and 16 (*M* = 7, s.d. = 4) for psychoticism, 0 and 18 (*M* = 9, s.d. = 6) for neuroticism and 9 and 22 (*M* = 16, s.d. = 4) for extraversion.

#### 2.2.2. Emotion recognition

**Figure 2.**
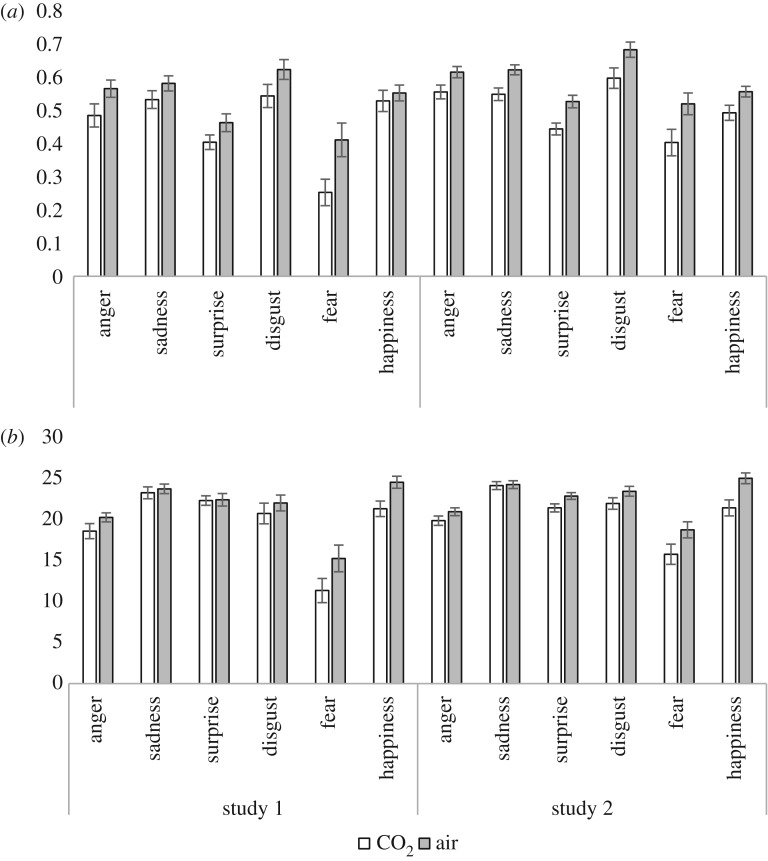
Mean (±s.e.) sensitivity (unbiased hit rate; *a*) and recognition accuracy (hits; *b*) for all six primary emotional expressions in study 1 (left) and study 2 (right).

A 2 × 6 ANOVA of hit data indicated a main effect of gas (*F*_1, 20_ = 20.59, *p *< 0.001, $\eta_{p}^{2}=0.51$), with fewer hits in the 7.5% CO_2_ condition (*M* = 19.6, s.d. = 2.5) compared with the air condition (*M* = 21.3, s.d. = 2.5). There was also a main effect of emotion (*F*_3.0, 60.8_ = 24.32, *p *< 0.001, $\eta_{p}^{2}=0.55$), which was qualified by a gas × emotion interaction (*F*_5, 100_ = 2.69, *p *= 0.025, $\eta_{p}^{2}=0.12$). Post hoc tests indicated fewer hits in the 7.5% CO_2_ condition for anger, disgust, fear and happiness (figure 2; electronic supplementary material, table S1).

#### 2.2.3. Emotion-specific sensitivity

There was evidence of lower recognition sensitivity in the 7.5% CO_2_ condition, compared with air, for all emotions (*p*s < 0.069) except happiness (*p* = 0.450) (figure 2; electronic supplementary material, table S2).

#### 2.2.4. Interpretation bias

One participant was identified as an outlier in both air and CO_2_ conditions and was removed from the analysis, leaving a sample size of 20 for analysis. Threshold scores on the 2AFC interpretation bias task were lower in the 7.5% CO_2_ condition (*M *= 6.7, s.d. = 1.0) compared with the air condition (*M *= 7.4, s.d. = 1.3) (*t*_19 _= 2.41, *p* = 0.027, *dz *= 0.54), indicating a greater bias towards seeing anger over happiness in the CO_2_ condition. When data from the participant identified as an outlier were included, this effect was unchanged (*p* = 0.018).

#### 2.2.5. Manipulation check

State anxiety (STAI State), negative affect (PANAS negative), SBP, DBP and HR were higher, and positive affect (PANAS positive) was lower, after CO_2_ inhalation compared with air (*p*s ≤ 0.095) (table 1).

**Table 1.** Change in state anxiety, affect and cardiovascular measures between 7.5% CO_2_ and air conditions in study 1 and study 2. STAI, Spielberger State-Trait Anxiety Inventory; PANAS, Positive and Negative Affect Schedule; SBP, systolic blood pressure; DBP, diastolic blood pressure; HR, heart rate.

	study 1 (*n* = 21)					study 2 (*n* = 43)			
	mean difference (s.d.)	effect size (*dz*)	95% CI	*p*-value		mean difference (s.d.)	effect size (*dz*)	95% CI	*p*-value
STAI State	23.4 (10.0)	2.3	18.8–27.9	<0.001		18.0 (11.2)	1.6	14.5–21.4	<0.001
PANAS negative	9.1 (5.3)	1.7	6.7–11.5	<0.001		7.1 (6.8)	1.0	5.1–9.2	<0.001
PANAS positive	−7.9 (6.4)	1.2	−10.8 to −5.0	<0.001		−4.2 (6.8)	0.6	−6.2 to 2.1	<0.001
SBP	7.2 (10.6)	0.7	2.4–12.0	0.006		12.3 (8.8)	1.4	9.7–15.0	<0.001
DBP	2.3 (6.0)	0.4	−0.4 to 5.0	0.095		0.2 (9.5)	0.0	−2.7 to 3.1	0.900
HR	15.0 (9.4)	1.6	10.7–19.3	<0.001		12.8 (14.7)	0.9	8.3–17.3	<0.001

## 3. Study 2

### 3.1. Methods

Study 2 was a direct replication of study 1, and all methods are identical. The protocol for this study was pre-registered on the Open Science Framework (https://osf.io/cr7ki/). The sample size was determined using data from study 1, which indicated an effect size of *dz *= 0.56 for the comparison of 2AFC threshold data between the 7.5% CO_2_ (*M *= 6.5, s.d. = 1.4) and air (*M *= 7.2, s.d. = 1.2) conditions (including data from the participant identified as an outlier). We chose 2AFC data as these indicated the smaller effect size, and therefore gave the most conservative sample size estimate. These data indicated that a sample of 44 participants would be required to achieve 95% power at an α-level of 5%. Data collection continued until this target was achieved. Participants were reimbursed £20 for taking part in the study.

### 3.2. Results

The data that form the basis of the results presented here are available from the University of Bristol Research Data Repository (http://data.bris.ac.uk/data/) (doi:10.5523/bris.108bq7qhjs5q81477djbcwnc2v).

#### 3.2.1. Characteristics of participants

Participants (*n* = 44; 50% male) were aged between 18 and 48 years (*M* = 25, s.d. = 6). STAI-Trait and ASI scores ranged between 22 and 52 (*M =* 32, s.d. = 7) and 4 and 33 (*M* = 14, s.d. = 7), respectively. EPQ-R scores ranged between 0 and 15 (*M* = 7, s.d. = 4) for psychoticism, 0 and 17 (*M* = 8, s.d. = 4) for neuroticism and 6 and 23 (*M* = 16, s.d. = 4) for extraversion.

#### 3.2.2. Emotion recognition

One participant was identified as an extreme outlier in the air condition, leaving a sample size of 43 for analysis. For hit data, there was a main effect of gas (*F*_1, 42_ = 20.26, *p *< 0.001, $\eta_{p}^{2}=0.33$) with fewer hits in the 7.5% CO_2_ condition (*M* = 20.7, s.d. = 2.9) compared with the air condition (*M* = 22.5, s.d. = 1.9) and a main effect of emotion (*F*_3.6,12.8.2_ = 18.49, *p *< 0.001, $\eta_{p}^{2}=0.31$). There was also a gas × emotion interaction (*F*_3.6,12.8.2_ = 3.92, *p *= 0.010, $\eta_{p}^{2}=0.09$). Post hoc tests indicated that there were fewer hits in the 7.5% CO_2_ condition for all emotions except sadness (figure 2; electronic supplementary material, table S1).

#### 3.2.3. Emotion-specific sensitivity

There was evidence of lower recognition sensitivity in the 7.5% CO_2_ condition, compared with air, for all emotions (*p*s < 0.010) (figure 2; electronic supplementary material, table S2).

#### 3.2.4. Interpretation bias

Data for one participant were lost due to computer malfunction, leaving a sample size of 43 for analysis. Threshold scores on the 2AFC task were lower in the 7.5% CO_2_ condition (*M *= 7.0, s.d. = 1.7) compared with the air condition (*M *= 7.5, s.d. = 1.8) (*t*_42_ = 1.76, *p* = 0.086, *dz *= 0.27). This replicated the finding from study 1, indicating a greater bias towards seeing anger over happiness in the 7.5% CO_2_ condition, although the effect was weaker in study 2.

#### 3.2.5. Manipulation check

State anxiety (STAI), negative affect (PANAS-negative), SBP and HR were higher, and positive affect (PANAS-positive) was lower, after CO_2_ inhalation compared with air (*p*s < 0.001). There was no effect of gas on DBP (*p* > 0.249) (table 1).

#### 3.2.6. Confusion matrices

Confusion matrices were calculated to show the percentage of categorizations made for each emotional expression (6AFC task data) during inhalation of air and CO_2_ (table 2). This allowed us to observe whether there were any changes in systematic categorization bias as a function of anxiety. There was a pattern of a bias in the categorization of fear as surprise, but overall, there was little evidence of systematic categorization biases and little difference between anxiety conditions.

**Table 2.** Confusion matrices of expression categorization in the 6AFC task during CO_2_ (*a*) and air (*b*) inhalations. Data (%) are combined across studies 1 and 2. Italicized numbers reflect ‘hits’ (i.e. trials in which response matched the emotion displayed).

emotion displayed	anger	sadness	surprise	disgust	fear	happiness
	(*a*) response (%) during CO_2_ inhalation					
anger	*64*	17	1	7	3	8
sadness	3	*79*	2	4	4	8
surprise	1	5	*72*	3	10	9
disgust	9	7	2	*72*	4	6
fear	1	3	45	2	*47*	2
happiness	2	12	5	5	5	*71*
	(*b*) response (%) during air inhalation					
anger	*69*	13	0	4	2	12
sadness	3	*80*	1	2	3	11
surprise	1	3	*76*	1	7	12
disgust	8	4	2	*75*	2	9
fear	0	2	38	1	*58*	1
happiness	1	7	2	5	3	*82*

## 4. Study 3

### 4.1. Methods

#### 4.1.1. Participants

Healthy volunteers were recruited using Amazon Mechanical Turk. Participants were reimbursed $2.25 for taking part in the study.

#### 4.1.2. Study design

The study examined the relationship between self-reported measures of state and trait anxiety, and performance on the 6AFC task via an online platform. The protocol for this study was pre-registered on the Open Science Framework (https://osf.io/qnrge/).

#### 4.1.3. Measures and materials

The 6AFC task was identical to that used in studies 1 and 2, except that the stimuli set was expanded to include both male and female faces of four ethnicities (European, African, South Asian, East Asian), and the task was shortened to comprise 96 trials (16 for each emotion, eight male and eight female), and participants were randomized to complete one of 16 versions of the task that presented one of four stimulus ethnicities and used one of four stimulus presentation times (125, 250, 500, 1000 ms). The STAI and the Patient Health Questionnaire (PHQ-9) [54] were used to assess trait and state anxiety and depressive symptoms, respectively. The online study also included a catch trial, designed to identify participants whose responses were not genuine (e.g. merely responding rapidly to complete the study) [55]. This consisted of a scale with options ranging from 1 (‘Very Rarely’) to 9 (‘Very Frequently’) and a small blue circle at the bottom of the page. Participants were instructed on-screen to click the small blue circle rather than selecting any of the scale items. Participants who responded incorrectly were excluded from our analyses.

#### 4.1.4. Procedure

After opting to take part in the study via the Amazon Mechanical Turk platform, participants were directed to an external website which hosted the study. Participants read an information sheet and provided informed consent, before providing sociodemographic information including age, sex, ethnicity (European, African, South Asian, East Asian, other), highest level of education attained (Some High School, High School Graduate or Some College, Bachelor Degree, Graduate or Professional Degree) and whether they were receiving any drug or behavioural treatments for mental health problems. Participants then completed the STAI and PHQ-9 questionnaires, followed by the 6AFC task. On completion of the 6AFC task, participants were presented with a debriefing page and informed that the study had ended.

#### 4.1.5. Statistical analysis

All statistical analyses were conducted using IBM SPSS Statistics for Windows (v. 21.0, IBM Corp.) and R (v. 3.2.2, www.R-project.org). Total hits were assessed for outliers using boxplots. Participant data were removed if scores were 3.0 times greater than the interquartile range, or if the participant failed the catch trial. Data were also assessed for normality using skewness and kurtosis statistics. We used linear regression to test for an association between STAI State score and total hits (to give an estimate of global emotion recognition ability). The same linear regression was conducted on hits and sensitivity scores (unbiased hit rate) for each individual emotional expression, both unadjusted, adjusted for sociodemographic characteristics, and additionally adjusted for STAI Trait score. A similar analysis was conducted for STAI Trait scores (adjusted for STAI State). The results of the analyses of PHQ-9 data will be reported elsewhere. Our planned sample size of *n* = 2000 provided us with 95% power to detect a correlation between STAI State score and 6AFC scores of *r* = 0.08 at an α-level of 5%.

### 4.2. Results

The data that form the basis of the results presented here are available from the University of Bristol Research Data Repository (http://data.bris.ac.uk/data/) (doi:10.5523/bris.112g2vkxomjoo1l26vjmvnlexj).

#### 4.2.1. Characteristics of participants

A total of 2006 participants were recruited. Of these, the data from 12 participants were excluded from the analysis for failing the catch trial, as specified in our protocol. We therefore analysed the responses from 1994 participants (48% male) aged between 18 and 75 years (*M* = 34, s.d. = 11). STAI State scores ranged between 20 and 80 (*M* = 36, s.d. = 12), and STAI Trait scores ranged between 20 and 78 (*M* = 40, s.d. = 13). The majority (*n* = 1632, 82%) were of European ancestry, and had a college degree or higher (*n* = 1021, 51%). A minority were currently undergoing pharmacological and/or behavioural treatment (*n* = 181, 9%).

#### 4.2.2. Global emotion recognition

Linear regression indicated that STAI State score was associated with reduced global emotion recognition accuracy (i.e. total hits) (unadjusted: *B* = −0.0671, 95% CIs = −0.1028 to −0.0315, *p* < 0.001; partially adjusted: *B* = −0.0735, 95% CIs = −0.1106 to −0.0364, *p* < 0.001; fully adjusted: *B* = −0.2554, 95% CIs = −0.3222 to −0.1887, *p* < 0.001).

#### 4.2.3. Emotion-specific sensitivity

Consistent with the findings from studies 1 and 2, there was little evidence that these results differed for specific emotions. Instead, there was evidence that state anxiety was associated with reduced recognition ability across all emotions for both hits and sensitivity. These results are shown in table 3. Analyses were conducted with and without participants whose total hits scores were 3.0 times greater than the interquartile range (*n* = 4), but these results did not differ, so results on the full sample are reported. As participant age and STAI State and Trait scores were positively skewed, we also conducted analyses using log-transformed variables, but these results did not differ and therefore, results using untransformed data are reported.

**Table 3.** Association between state anxiety and global emotion recognition (total hits), and emotion-specific recognition sensitivity and hits (*n* = 1994).

	unadjusted			partially adjusted^a^			fully adjusted^b^		
	*B*	95% CI	*p*-value	*B*	95% CI	*p*-value	*B*	95% CI	*p*-value
total hits	−0.0671	−0.1028 to −0.0315	<0.001	−0.0735	−0.1106 to −0.0364	<0.001	−0.2554	−0.3222 to −0.1887	<0.001
	hits by emotion								
anger	−0.0059	−0.0161 to 0.0043	0.260	−0.0076	−0.0183 to 0.0030	0.161	−0.0386	−0.0578 to −0.0193	<0.001
sadness	−0.0107	−0.0205 to −0.0009	0.033	−0.0138	−0.0240 to −0.0036	0.008	−0.0529	−0.0713 to −0.0344	<0.001
surprise	−0.0069	−0.0149 to 0.0011	0.090	−0.0062	−0.0146 to 0.0022	0.146	−0.0186	−0.0338 to −0.0034	0.017
disgust	−0.0172	−0.0292 to −0.0052	0.005	−0.0196	−0.0321 to −0.0071	0.002	−0.0550	−0.0777 to −0.0324	<0.001
fear	−0.0142	−0.0269 to −0.0016	0.028	−0.0167	−0.0299 to −0.0035	0.013	−0.0640	−0.0879 to −0.0401	<0.001
happiness	−0.0122	−0.0213 to −0.0031	0.008	−0.0095	−0.0190 to 0.0000	0.049	−0.0264	−0.0436 to −0.0092	0.003
	sensitivity by emotion								
anger	−0.0004	−0.0011 to 0.0003	0.274	−0.0005	−0.0012 to 0.0002	0.157	−0.0034	−0.0047 to −0.0021	<0.001
sadness	−0.0006	−0.0012 to 0.0000	0.060	−0.0007	−0.0014 to −0.0001	0.024	−0.0031	−0.0042 to −0.0019	<0.001
surprise	−0.0005	−0.0010 to −0.0001	0.025	−0.0005	−0.0010 to −0.0001	0.030	−0.0022	−0.0031 to −0.0013	<0.001
disgust	−0.0012	−0.0020 to −0.0005	0.001	−0.0013	−0.0021 to −0.0005	0.001	−0.0037	−0.0051 to −0.0022	<0.001
fear	−0.0009	−0.0016 to −0.0001	0.024	−0.0011	−0.0018 to −0.0003	0.008	−0.0039	−0.0053 to −0.0025	<0.001
happiness	−0.0008	−0.0013 to −0.0003	0.003	−0.0008	−0.0013 to −0.0002	0.006	−0.0024	−0.0033 to −0.0014	<0.001

^a^Adjusted for sociodemographic characteristics (age, sex, ethnicity, level of education, treatment status).

^b^Additionally adjusted for STAI Trait score.

Of note, we observed that STAI Trait score was associated with *increased* global emotion recognition accuracy (i.e. total hits) when analyses were fully adjusted, including STAI State score (unadjusted: *B* = 0.0078, 95% CIs = −0.0255 to 0.0412, *p* = 0.645; partially adjusted: *B* = 0.0042, 95% CIs = −0.0312 to 0.0397, *p* = 0.815; fully adjusted: *B* = 0.2074, 95% CIs = 0.1438 to 0.2710, *p* < 0.001) and *increased* emotion recognition sensitivity when analyses were fully adjusted including STAI State score across emotions (unadjusted: *B*s = −0.0002 to 0.0006, 95% CIs = −0.0009 to 0.0013, *p*s > 0.053; partially adjusted: *B*s = −0.0002 to 0.0006, 95% CIs = −0.0010 to 0.0013, *p*s > 0.099; fully adjusted: *B*s = 0.0018–0.0033, 95% CIs = 0.0009–0.0046, *p*s < 0.001). There was no clear evidence that these results differed for specific emotions. These results are shown in table 4.

**Table 4.** Association between trait anxiety and global emotion recognition (total hits), and emotion-specific hits and recognition sensitivity (*n* = 1994).

	unadjusted^a^			partially adjusted^a^			fully adjusted^b^		
	*B*	95% CI	*p*-value	*B*	95% CI	*p*-value	*B*	95% CI	*p*-value
total hits	0.0078	−0.0255 to 0.0412	0.645	0.0042	−0.0312 to 0.0397	0.815	0.2074	0.1438–0.2710	<0.001
	hits by emotion								
anger	0.0055	−0.0040 to 0.0150	0.258	0.0046	−0.0055 to 0.0147	0.373	0.0353	0.0169–0.0536	<0.001
sadness	0.0055	−0.0037 to 0.0146	0.242	0.0024	−0.0073 to 0.0122	0.623	0.0445	0.0269–0.0620	<0.001
surprise	−0.0019	−0.0093 to 0.0056	0.623	−0.0007	−0.0086 to 0.0073	0.869	0.0141	−0.0004 to 0.0286	0.056
disgust	−0.0011	−0.0123 to 0.0101	0.846	−0.0034	−0.0154 to 0.0085	0.575	0.0404	0.0188–0.0620	<0.001
fear	0.0049	−0.0070 to 0.0167	0.421	0.0030	−0.0096 to 0.0156	0.640	0.0539	0.0311–0.0767	<0.001
happiness	−0.0050	−0.0135 to 0.0035	0.248	−0.0017	−0.0108 to 0.0073	0.705	0.0193	0.0029–0.0356	0.021
	sensitivity by emotion								
anger	0.0006	0.0000–0.0013	0.053	0.0006	−0.0001 to 0.0013	0.099	0.0033	0.0020–0.0045	<0.001
sadness	0.0004	−0.0002 to 0.0009	0.201	0.0002	−0.0004 to 0.0008	0.447	0.0027	0.0016–0.0038	<0.001
surprise	0.0001	−0.0004 to 0.0005	0.686	0.0001	−0.0004 to 0.0006	0.619	0.0018	0.0010–0.0027	<0.001
disgust	−0.0002	−0.0009 to 0.0005	0.611	−0.0002	−0.0010 to 0.0005	0.536	0.0027	0.0013–0.0040	<0.001
fear	0.0003	−0.0004 to 0.0010	0.385	0.0001	−0.0006 to 0.0009	0.710	0.0032	0.0019–0.0046	<0.001
happiness	−0.0001	−0.0006 to 0.0004	0.605	0.0000	−0.0006 to 0.0005	0.864	0.0018	0.0009–0.0028	<0.001

^a^Adjusted for sociodemographic characteristics (age, sex, ethnicity, level of education, treatment status).

^b^Additionally adjusted for STAI State score.

## 5. Discussion

Our results indicate that state anxiety causally impairs emotion recognition, and induces interpretation bias. In two experimental studies that directly manipulated state anxiety, we found lower emotion recognition accuracy (hits) and emotion-specific sensitivity (unbiased hit rate), following anxiety induction. For recognition accuracy (hits), there was evidence of a gas by emotion interaction in study 1 indicating poorer recognition during 7.5% CO_2_ on a subset of emotions (fear, happiness, anger and disgust). However, this effect did not replicate in study 2, and the overall pattern of the data indicates reduced numbers of hits across all emotions in both studies. In addition, there was evidence of lower sensitivity (unbiased hit rate) to all emotions (with exception of happiness in study 1). Taken together, these findings suggest that state anxiety leads to a global, rather than emotion-specific, impairment of emotion recognition. These findings were supported by the large observational study. In addition to changes in recognition, there was also evidence of increased interpretation biases during state anxiety, with an increased tendency in perceiving anger/decreased tendency to perceive happy in ambiguous angry–happy facial morphs in both study 1 and study 2.

There was no evidence that state anxiety leads to heightened recognition of fearful emotional expressions, as has been previously reported in high trait anxious samples [19]. One interpretation of these findings is that state and trait anxiety have differential effects on emotional processing, and therefore, we should not expect state manipulations to necessarily produce findings consistent with trait comparisons. In support, Bourne & Vladeanu [56] report different patterns of neural activation in response to emotional faces among individuals who were either high trait anxious or had high self-reported state anxiety in response to a social stressor.

Comparisons between state and trait anxiety are complicated, however, by inconsistencies in the trait anxiety literature, with some studies reporting emotion-specific benefits and others reporting global detriments. This ambiguity may be in part due to differences in either the clinical diagnoses of anxiety (e.g. social anxiety disorder, generalized anxiety disorder) or the degree of trait anxiety in non-clinical samples. There is greater consistency in attentional compared with recognition outcomes. For example, several studies report emotion-specific (e.g. fear, anger) attentional biases indicative of enhanced threat processing. By contrast, recognition outcomes are mixed, which may also be due to differential use of recognition accuracy (hits) versus recognition sensitivity (unbiased hit rate) measures. Unbiased hit rates take account of both accuracy and false alarms. A processing (threat) bias would present as improved fear processing if accuracy is the dependent variable, but lower sensitivity (due to high false alarm rate) if unbiased hit rate is used. Most studies in this area have used accuracy outcomes, which makes it difficult to determine whether the effect is driven by a genuine improvement in recognition of fear versus a general bias towards perceiving fear regardless of whether it is present.

The current studies did not find evidence of improvements in fear recognition accuracy during heightened *state* anxiety, which is also inconsistent with previous research using a cognitive stressor (threat of shock) [32–34]. Instead, 7.5% CO_2_-induced state anxiety generally led to decreases in sensitivity across all emotions. We postulate that the lack of emotion-specific effects (particularly enhanced fear processing) may be because the CO_2_ anxiety manipulation is not explicitly valenced (i.e. lacks social, cognitive or environmental threat). Instead, these data suggest that the physiological component of state anxiety induces a general processing detriment, and additional cognitive or social components may be required to induce specific threat-related processing. This warrants further investigation, but offers important insight into the nature of the effects of subcomponents of state anxiety on emotional processing.

An alternative explanation for the lack of fear-specific effects is that participants were not highly trait anxious, and this may be an important mediator of fear processing during high anxiety states. For example, attentional control theory (ACT) emphasizes the importance of interactive effects of trait anxiety and situational stress, which together determine the anxious ‘state’ of the individual. While state anxiety due to an external stressor may lead to global deficits (through reductions in processing efficiency), ACT predicts that the attentional capture of threat-related stimuli is realized when the individual experiences subjective worry or self-preoccupation, which is greater in trait anxiety [57]. It is noteworthy that in the observational study, trait anxiety was associated with improved emotion recognition when we adjusted for state anxiety. This suggests that the effects of trait anxiety may be mediated by differences in state anxiety, and this could also explain the ambiguity in the trait literature. These findings need to be replicated and extended; however, they highlight the importance of exploring this interaction experimentally in future research.

The 2AFC findings indicated that state anxiety also leads to an increased bias in perceiving emotionally ambiguous faces (anger–happy morphs) as angry. However, as only one emotional continuum was used, we cannot determine whether this was due to a specific increase in bias towards seen anger (perhaps driven by an increase in negative threat perception) or to a decrease in interpreting the faces as happy. It is also unclear whether similar effects would be observed for other negative (but non-threatening) emotions such as sadness. The limited time available for testing during 7.5% CO_2_ inhalations (i.e. up to 20 min) meant we were unable to include more than one emotional continuum. This should be investigated further in future research.

The 2AFC data suggested that state anxiety increases negative bias towards anger (and away from happiness). In support, there were also increased false alarms (6AFC task) for anger during 7.5% CO_2_ inhalation (raw false alarm data available online). Arguably, a bias in processing of an emotion would also result in greater numbers of hits; however, we did not observe this during 7.5% CO_2_ inhalation in study 1 or study 2. This may indicate that the 2AFC outcomes (i.e. increased anger bias during heightened state anxiety) were driven by changes in happiness processing rather than anger or that the bias to anger is driven by increases in false alarms (i.e. erroneously identifying other emotions as anger) rather than correct identification of anger *per se*.

There are some limitations that should be considered when interpreting these results. First, the samples in the experimental studies consisted largely of young undergraduate students who may not be representative of the general population. However, the within-subjects nature of the experimental design raises confidence in our conclusions, as does the concordance between the results of our experimental and observational studies. Second, we only included one task that assessed emotion recognition bias that used a happy–angry morph sequence. This was due to the limited time available within the inhalation procedure. It would be valuable to explore the effects of anxiety on other ambiguous expressions. Third, we did not include a negative control task in our experimental studies, again due to time constraints during the inhalation procedure, so we cannot rule out the possibility that the effect of state anxiety on emotion recognition sensitivity is due to a global performance deficit.

## 6. Conclusion

The findings from these studies suggest that state anxiety causes a global decrease in emotion recognition and a bias towards identifying anger in ambiguous facial expressions. There was no evidence of increased recognition of fear, as has been associated with trait anxiety (albeit inconsistently). We are unable to ascertain from these data whether the effects reported here are specific to emotional face processing or represent more general impairments in cognitive processing. However, previous studies using the same CO_2_ challenge have consistently shown increased threat processing across a variety measures, implying that the lack of emotion-specific effects was not due to a lack of threat processing *per se*. As noted above, ACT suggests state anxiety may lead to global deficits in performance in the absence of trait anxiety, which is what we observed in studies 1 and 2 using unselected samples. It is plausible that high trait anxiety mediates emotional processing during state anxiety. For example, a dispositional tendency to perceive threat may be more likely to influence emotion-specific processing (such as increased bias to fearful faces) that is not evident in non-trait anxious samples. However, it is noteworthy that trait anxiety was associated with improved performance in study 3 when state anxiety was adjusted for, indicating that emotion processing deficits may be mediated by state anxiety in anxious groups, although this requires replication in anxious samples. This would suggest that clinical samples may have problems with social interaction during heightened state anxiety. This would be expected to lead to a negative cycle of social situations inducing anxiety, and fits with the Harmer model of emotional processing in psychiatric disorder [13], in which aberrant emotional processing plays a role in disorder maintenance. Therefore, future research should investigate the interactive effects of state and trait anxiety on emotional face processing. It would be important to identify whether the trait effect (of heightened recognition of fearful faces) can be replicated and whether it is amplified by increases in state anxiety.

## Supplementary Material

Supplementary Table S1

## Supplementary Material

Supplementary Table S2

## Data accessibility

The protocols for studies 2 and 3 were pre-registered on the Open Science Framework and are available at the following links, respectively: https://osf.io/cr7ki/, https://osf.io/qnrge/. The data that form the basis of the results presented in this paper are available from the University of Bristol Research Data Repository (http://data.bris.ac.uk/data/). The DOIs for the records associated with these datasets are: (doi:10.5523/bris.tdkhpfjk64ip1o86m4tq9e306) (study 1), (doi:10.5523/bris.108bq7qhjs5q81477djbcwnc2v) (study 2), (doi:10.5523/bris.112g2vkxomjoo1l26vjmvnlexj) (study 3) [58].
