# Supplementary material for: State anxiety and emotional face recognition in healthy volunteers
Source: R Soc Open Sci. 2017 May 31;4(5):160855. doi: 10.1098/rsos.160855 (PMC5451788; doi:10.1098/rsos.160855)
Supplement: Supplementary Table S1 [file rsos160855supp1.docx]

Supplementary Table S1. Post-hoc paired-sample t-test results of hit data from studies one and two.

|  | Study one (*n* = 21) | | | | | Study two (*n* = 43) | | | | |
| --- | --- | --- | --- | --- | --- | --- | --- | --- | --- | --- |
|  | Mean difference (*SD*) | *t* | 95% CI | *p*-value | Effect size (*dz*) | Mean difference (*SD*) | *t* | 95% CI | *p*-value | Effect size (*dz*) |
| Anger | -1.67 (4.04) | -1.89 | -3.51 to 0.17 | 0.073 | 0.41 | -1.09 (4.20) | -1.71 | -2.39 to 0.20 | 0.095 | 0.26 |
| Sadness | -0.47 (2.60) | -0.84 | -1.68 to 0.71 | 0.411 | 0.18 | -0.12 (2.63) | -0.29 | -0.93 to 0.69 | 0.773 | 0.04 |
| Surprise | -0.01 (3.83) | -0.11 | -1.84 to 1.65 | 0.910 | 0.02 | -1.44 (2.78) | -3.40 | -2.30 to -0.59 | 0.001 | 0.52 |
| Disgust | -1.29 (3.33) | -1.77 | -2.80 to 0.23 | 0.092 | 0.39 | -1.49 (4.49) | -2.18 | -2.87 to -0.11 | 0.035 | 0.33 |
| Fear | -3.91 (5.95) | -3.01 | -6.61 to -1.20 | 0.007 | 0.66 | -2.98 (7.72) | -2.53 | -5.35 to -0.60 | 0.015 | 0.39 |
| Happiness | -3.24 (4.49) | -3.01 | -5.49 to -0.99 | 0.007 | 0.66 | -3.61 (4.32) | -5.48 | -4.93 to -2.28 | <0.001 | 0.84 |

Abbreviations: SD – standard deviation (of mean difference)
