# Supplementary material for: State anxiety and emotional face recognition in healthy volunteers
Source: R Soc Open Sci. 2017 May 31;4(5):160855. doi: 10.1098/rsos.160855 (PMC5451788; doi:10.1098/rsos.160855)
Supplement: Supplementary Table S2 [file rsos160855supp2.docx]

Supplementary Table S2. Paired-sample t-test results of sensitivity (unbiased hit rate) data from studies one and two.

|  | Study one (*n* = 21) | | | | | Study two (*n* = 43) | | | | |
| --- | --- | --- | --- | --- | --- | --- | --- | --- | --- | --- |
|  | Mean difference (*SD*) | *t* | 95% CI | *p*-value | Effect size (*dz*) | Mean difference (*SD*) | *t* | 95% CI | *p*-value | Effect size (*dz*) |
| Anger | -0.081 | -2.26 | -0.156 to -0.006 | 0.035 | 0.49 | -0.060 | -2.72 | -0.104 to -0.016 | 0.009 | 0.42 |
| Sadness | -0.049 | -1.93 | -0.101 to -0.004 | 0.068 | 0.42 | -0.074 | -3.83 | -0.113 to -0.035 | <0.001 | 0.58 |
| Surprise | -0.059 | -2.83 | -0.103 to -0.016 | 0.010 | 0.62 | -0.083 | -4.62 | -0.119 to -0.047 | <0.001 | 0.70 |
| Disgust | -0.079 | -2.99 | -0.135 to -0.024 | 0.007 | 0.65 | -0.086 | -3.64 | -0.133 to -0.038 | 0.001 | 0.55 |
| Fear | -0.159 | -3.69 | -0.249 to -0.069 | 0.001 | 0.81 | -0.117 | -2.99 | -0.197 to -0.038 | 0.005 | 0.46 |
| Happiness | -0.024 | -0.77 | -0.089 to 0.041 | 0.450 | 0.17 | -0.063 | -3.12 | -0.105 to -0.022 | 0.003 | 0.48 |

Abbreviations: SD – standard deviation (of mean difference)
